# The Case for Proteomics and Phospho‐Proteomics in Personalized Cancer Medicine

**DOI:** 10.1002/prca.201800113

**Published:** 2019-03-29

**Authors:** Sophia Doll, Florian Gnad, Matthias Mann

**Affiliations:** ^1^ Department of Proteomics and Signal Transduction Max Planck Institute of Biochemistry 82152 Martinsried Germany; ^2^ NNF Center for Protein Research Faculty of Health Sciences University of Copenhagen Copenhagen Denmark; ^3^ Department of Bioinformatics and Computational Biology Cell Signaling Technology Inc 01923 Danvers MA USA

**Keywords:** mass spectrometry, clinical proteomics, oncology

## Abstract

The concept of personalized medicine is predominantly been pursued through genomic and transcriptomic technologies, leading to the identification of multiple mutations in a large variety of cancers. However, it has proven challenging to distinguish driver and passenger mutations and to deal with tumor heterogeneity and resistant clonal populations. More generally, these heterogeneous mutation patterns do not in themselves predict the tumor phenotype. Analysis of the expressed proteins in a tumor and their modification states reveals if and how these mutations are translated to the functional level. It is already known that proteomic changes including posttranslational modifications are crucial drivers of oncogenesis, but proteomics technology has only recently become comparable in depth and accuracy to RNAseq. These advances also allow the rapid and highly sensitive analysis of formalin‐fixed and paraffin‐embedded biobank tissues, on both the proteome and phosphoproteome levels. In this perspective, pioneering mass spectrometry‐based proteomic studies are highlighted that pave the way toward clinical implementation. It is argued that proteomics and phosphoproteomics could provide the missing link to make omics analysis actionable in the clinic.

## 1 Introduction

Cancer, “The Emperor of all Maladies,”^1^ was responsible for nine million deaths worldwide in 2018, despite harsh treatments from which many patients do not derive any benefit.^2^ The poor clinical outcome for most cancer types originates from a diverse array of factors, including late diagnosis, tumor heterogeneity, metastasis, lack of targeted treatment options and resistance to therapy, tumor recurrence, and a failure to translate preclinical breakthroughs into meaningful patient benefit.

The rapidly decreasing costs of next‐generation sequencing (NGS) technologies^3^ have triggered an explosion of genomic data, improving our overall understanding of cancer. The Cancer Genome Atlas (TCGA)^4^ and the International Cancer Genome Consortium (ICGC)^5^ have surpassed the 1000 Genomes Project by sequencing thousands of tumors across different cancer types. Comprehensive genetic profiling of tumor samples has uncovered novel oncogenes and tumor suppressor genes by comparing their mutation frequencies with the background mutation rate,^6^ or by detecting mutation profiles with significant bias toward certain mutation types such as non‐silent,^7^ functional,^8^ or hotspot^9^ mutations. In turn, this has transformed the target selection process in many pharmaceutical companies and led to the development of personalized sequencing tests that are increasingly being offered in a clinical setting. For example, TCGA data revealed an enormous mutation burden in epigenetic regulators,^10^ including the SWI/SNF complex subunits protein polybromo‐1 (PBRM1) and AT‐rich interactive domain‐containing protein 1A (ARID1A), which are mutated in approximately one third of clear cell renal cell carcinomas^11^ and endometrial carcinomas.^12^ Similarly, a significant proportion of bladder cancer patients showed mutations in multiple genes including histone‐lysine *N*‐methyltransferase 2D (MLL2), cyclin‐dependent kinase inhibitor 1 (CDKN1A), general transcription and DNA repair factor IIH helicase subunit XPD (ERCC2), or cohesin subunit SA‐2 (STAG2) previously not directly linked to this disease.^13^ It has been estimated that a comprehensive catalog of cancer genes requires the analysis of ≈2000 tumors for each of at least 50 tumor types, corresponding to 100 000 tumors.^14^ Given the rate of tumor genome sequencing, the available NGS data are already close to this magnitude.

In many cases, however, the involvement of these mutated genes was already known before the sequencing boom.^15^ Overall, NGS of the tumor genome identified actionable genes in about one third of cancer patients, but only a fraction of patients showed clinical benefit.^16^, ^17^ Extending NGS to the transcript level (RNAseq) should provide a more comprehensive picture of the tumor landscape. Applied to the detection of gene fusions and to the differential expression, it has elucidated potential biomarkers and previously unknown biology in combination with DNA sequencing.^18^, ^19^ In this way, exome sequencing uncovered mutations in kelch‐like ECH‐associated protein 1 (KEAP1) in ≈20% of lung adenocarcinomas,^20^ while matching RNA sequencing revealed a constitutively activated transcriptional response of 27 genes as a potential biomarker signature for KEAP1 mutant tumors.^21^

This illustrates that the limitations of genomics and transcriptomics restrict our ability to fully comprehend the pathophysiology and complexity of cancer. Instead, panomics, the integration of multiple “omic” approaches may be better able to decipher causal relationships.^22^ Furthermore, it has become clear that the development and complexity of cancer cannot be explained simply by genetic alterations and transcriptional changes alone. Instead, the investigation of proteins and their posttranslational modifications (PTMs)—the driving molecular entities in our cells—is necessary to provide insight into perturbed disease states. Transcript expression does not necessarily correlate with protein abundance,^23^, ^24^ and the action and dynamics of proteins including PTM‐mediated processes, subcellular localization, and regulatory mechanisms dictating protein accumulation or degradation are invisible to genomics‐based approaches. Thus, developing methods to directly measure and annotate protein abundance, interaction, localization, and modification states may offer new insights into the pathophysiology of tumors and aid in the identification and validation of novel drug targets.

Protein mass spectrometry (MS) has emerged as the technology of choice for large‐scale and unbiased proteomic analyses.^25^ Recent groundbreaking technological improvements now allow the analysis of the proteome at a large scale and with short turnaround times.^25^ These advances enable the measurement of nearly complete proteomes, as more than 12 000 proteins and more than 10 000 sites of different PTMs can be identified and quantified in complex cellular systems, including clinical samples.^26^, ^27^, ^28^, ^29^, ^30^ Proteomics has also become highly sensitive, requiring minute sample amounts (<10–100 µg) in cell culture as well as in clinical samples.^31^, ^32^ Given these technological advances, MS‐based proteomics has already been applied to clinical cancer cohorts.^33^, ^34^, ^35^ However, these workflows were highly specialized and not meant to be used in the clinic.

Here, we advocate the broad adoption of proteomics in oncological research and clinical settings. We believe that robust, highly sensitive, and specific personalized proteomic technology can be developed to profile tumor‐specific expression of proteins and PTMs. By combining proteomic data with genomic and clinical data, the ultimate aim is to generate a personalized panomics profile for each patient to better inform treatment decisions.^22^ Large initiatives such as the Obama Precision Medicine program^36^, ^37^ highlight the importance of taking individual molecular variability into account. In this way, patients who are most likely to respond and benefit from a given treatment may be better distinguishable from those who will only suffer from detrimental side effects.^38^, ^39^

## 2 Challenges of Translating Genomics Data into Clinical Use

The genomic interrogation of diverse tumor types has led to the discovery of the genetic basis of their underlying abnormalities. In the classical case of the Philadelphia chromosome, a translocation causes chronic myeloid leukemia through the constitutively active BCR‐ABL fusion—one of the first genetic alterations that could be linked to pathophysiology.^40^ Blocking this molecular perturbation with tyrosine kinase inhibitors, such as Imatinib, was a pioneering achievement for targeted therapy in cancer.^40^ Other successful implementations of targeted therapies, resulting from their genetic characterization, include blocking receptor tyrosine‐protein kinase erbB‐2 (HER2) in breast cancers with Herceptin,^41^ inhibiting anaplastic lymphoma kinase (ALK) in lymphoma with Crizotinib,^42^ or interrupting epidermal growth factor receptor (EGFR) activity in lung cancer with Gefinitib.^43^ Although most of these developments predate modern sequencing technology, NGS can be efficiently utilized as a discovery and diagnostic tool on which targeted therapies can be based. The comprehensiveness of NGS technologies routinely allows the detection of the entire spectrum of genetic abnormalities. This is important as tumors may either result from a single, initial mutation, such as the GTPase K‐RAS^G12D^ or Phosphoinositide‐3‐kinase PIK3CA^H1057R^ mutation in the PI3 kinase pathway^44^ or rely on the concerted action of many genetic alterations for uncontrolled cell growth and proliferation.

Despite the remarkable success of genomic‐based approaches, the majority of tumors do not present a clearly actionable genetic alteration. Furthermore, patients often do not respond to such targeted genetic therapies.^45^, ^46^ In many cases, poor or no responses can be traced back to heterogeneity of the tumors. This subclonal architecture can arise through intercellular genetic instability, followed by selective outgrowth of clones that have a phenotypic advantage during treatment and within the given micro‐environmental context.^47^ Deep sequencing from single cells is a promising technology to decipher the complexity of tumors.^48^ However, the resulting complex and heterogeneous mutation spectrum usually presents even greater challenges in selecting a treatment because it is difficult to predict whether the presence of genetic abnormalities will translate into downstream levels of gene expression and into the phenotype of cells and tissues. Obviously, genomic and transcriptomic changes can only have an impact on the phenotype if they are indeed translated into the proteomic level.

At the methodological level, an obstacle to using RNAseq data in the clinic is the preservation of tumor biopsies as formalin‐fixed and paraffin‐embedded (FFPE), where RNA is unstable resulting in poor sample quality.^49^ Although alternatives exist, FFPE is by far the most common storage modality for tissue biopsies due to its capabilities for long preservation and its amenability to H&E staining and immunohistochemistry. It is estimated that about half a billion archived FFPE cancer tissue samples exist to date and this number is rapidly rising.^50^ These immense archives of material present an invaluable resource for studying the underlying molecular mechanisms of cancer, testing known biomarkers and uncovering new ones.

## 3 The Promise of Proteomics

In almost all cases, proteins are the functional biological entities in cells, working in concert with each other as molecular machines or in pathways, ensuring that each cell performs its specific biological functions. Drugs targeting mitogen‐activated protein kinase 1 (MAPK), PI3K, serine/threonine‐protein kinase B‐raf (BRAF), vascular endothelial growth factor (VEGF), ALK, and EGFR inhibit their targets directly at the protein level—and not at the gene level.^15^ The phosphorylation intensities of associated substrates are commonly used as a proxy to detect aberrant kinase activities. Phosphoproteomics measures the identity and quantity of tens of thousands of phosphorylation sites, serving as an ultimate read‐out of the activity of kinases and altered signaling pathways, which are among the most important alterations in oncogenic transformation. Consequently, most of the laboratory tests performed for diagnosis and therapy are in general based on proteins.^51^ In pathology, immunohistochemistry to stain proteins in cancer tissues is still the mainstay for tumor classification and decisions on therapy. Thus, protein analysis plays a central role in current clinical practice and diagnostics. Proteins can be localized in different subcellular compartments, exhibit regulated degradation, and can be further modified by PTMs. These events happen downstream of gene expression, and therefore transcript abundance does not necessarily correlate with protein abundance and activity.^23^ It is thus important to directly measure protein abundance and modifications to characterize the disease status.

On a practical level, proteins are more stable than oligonucleotides, especially RNA.^52^, ^53^ This makes profiling of proteins inherently more robust and forgiving of sample quality. Today, protein abundance is determined in high‐throughput platforms that analyze proteins in automated antibody‐based workflows. However, this is almost always restricted to individual and already known proteins, negating the possibility to uncover novel biomarkers. In contrast, MS‐based proteomics measures thousands of proteins at once (ideally the entire expressed proteome) and is unbiased in this sense. Furthermore, this technology does not depend on protein epitopes being preserved, unlike antibody‐based methods.

Taking advantage of the stability and ease‐of‐handling of both unmodified or posttranslationally modified proteins, extraction from FFPE material is possible in a robust manner for MS‐based analysis. The comparison of FFPE to fresh tissues did not reveal major quantitative or qualitative differences at the protein or PTM level, including phosphorylation and even 30‐year old FFPE tissues have been analyzed successfully.^54^, ^55^ Thus MS‐based proteomics holds great promise for discovering new biomarkers and improving prognostic and predictive power for clinicians.

## 4 Recent Technological Advances of Mass Spectrometry‐Based Proteomics

Because of its high specificity, sensitivity, and accuracy, MS has emerged as the technology of choice for large‐scale and unbiased proteomic analyses, presenting a promising solution for the challenges described.^25^

**Figure 1:**
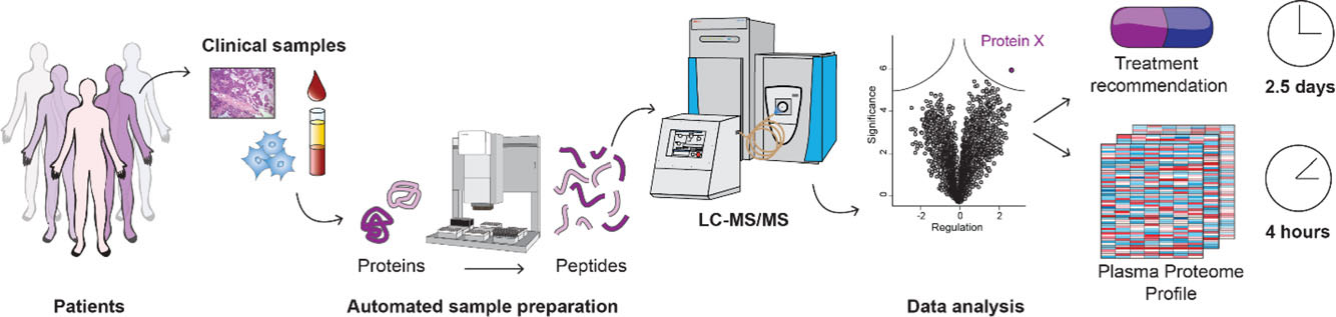
MS‐based proteomic workflow. Cancer samples, such as FFPE tissues, blood plasma, or cells are analyzed using automated sample preparation methods and analyzed by high resolution MS. The generated proteomic data are analyzed using sophisticated but automated bioinformatic workflows resulting in a set of several thousand identified and quantified proteins.

A typical MS‐based proteomic workflow first requires the lysis of the biological source of material, in order to efficiently extract proteins (**Figure** 1). This step incorporates heating to increase lysis efficiency and denature proteins, and is particularly important for de‐crosslinking FFPE samples. Cysteines of the extracted proteins are then reduced and alkylated to disrupt disulfide bridges prior to digestion. Lys‐C in combination with trypsin are generally the enzymes of choice due to their high cleavage specificity to lysines and arginines, yielding a defined set of identifiable peptides as search space for subsequent search algorithms. These sample preparation steps can now be automated and performed in a single device (“in‐StageTip” method^56^), considerably reducing sample preparation time, contamination and material loss while increasing reproducibility and throughput. The investigation of PTMs using MS requires additional enrichment steps and hence larger starting materials (by a factor 10–100) as PTM sites are generally substoichiometrically occupied. Common enrichment strategies use affinity purification based on charge properties or antibody recognition.^57^, ^58^ Phosphorylation is one of the most important and commonly studied PTMs (see statistics of PhosphoSitePlus; www.phosphosite.org).^59^ This has prompted the development of highly effective protocols, such as the EasyPhos method.^60^, ^61^ High‐throughput enrichment methods using robotic assistance are also becoming possible.^62^ For instance, the AssayMAP robotic technology (Agilent Technologies) enables the enrichment of phosphopeptides from 96 samples in only 1 h.^63^

Peptides are loaded onto a C_18_‐bead filled capillary column and then subjected to high performance liquid chromatography (HPLC) separation, before electrospray ionization and transfer to the mass spectrometer. For maximum sensitivity, column diameters and flowrates are chosen to be as low as possible, which can compromise robustness of the measurement. Novel LC systems have significantly increased reliability and reduced times between runs to a few minutes.^64^ Hence, the measurement time of a mass spectrometer, which remains the most cost‐intensive part of the workflow, can be used more efficiently.

The MS peptide data can be acquired using different methods, such as data‐dependent acquisition (DDA) modes in which the mass spectrometer automatically fragments the top most abundant peptide peaks in each scan. Currently, Orbitrap‐based mass spectrometers are dominant, although in our group the combination of trapped ion mobility with time of flight MS has recently shown much promise.^65^ Apart from the sensitivity of a measurement, which determines the needed input material, another crucial parameter is the dynamic range—meaning the ability to measure low abundance proteins in the presence of very highly abundant ones. For instance, in muscle tissue, protein copy numbers can range from tens to more than many million per cell.^28^, ^51^, ^66^ New MS acquisition methods, such as “BoxCar,” can extend the accessible dynamic range at least tenfold.^67^

Data‐independent acquisition (DIA) measurements such as “sequential window acquisition of all theoretical mass spectra” (SWATH) isolate multiple peptides within a specific *m*/*z* range.^68^, ^69^ Selected peptides are then co‐fragmented and measured simultaneously. The resulting complex fragment spectra can be analyzed using appropriate bioinformatics methods.^70^ A principal attraction of DIA is that it reduces the “missing value problem,” which is especially pertinent in clinical studies. Recent technological advances have made DIA increasingly competitive, featuring high dynamic range, reproducible and accurate protein quantification with CVs reaching 10% or below.^71^, ^72^

Targeted proteomic methods are used to analyze a limited set of predefined peptides with high reproducibility and specificity. They come in different flavors, including single, multiple, and parallel reaction monitoring (SRM, MRM, PRM),^73^, ^74^ but by their nature they are not applicable to the discovery of novel biomarkers.

The advances in MS instrumentation go hand in hand with the development of required software. These tools have become increasingly powerful and user‐friendly, enabling comprehensive analysis of large proteomic datasets and their integration with other “omics” data layers.^75^

Together, these technological advances have drastically improved all steps of today's MS workflow, and have enabled the characterization of nearly complete proteomes in recent years.^25^ The identification and quantification of more than 10 000 different proteins from minute amount of material, including FFPE tissues, is becoming feasible.^33^, ^76^, ^77^, ^78^ Thus, MS‐based proteomics now enables robust, reproducible, and in‐depth profiling of the proteome with fast turnaround times, for instance requiring only 4 h for blood plasma proteomics and two and a half days for tissues.^79^, ^80^

## 5 MS‐Based Proteomics in Cancer Discovery

As the sensitivity of MS has substantially increased in recent years, the technology has begun to enable the elucidation of previously unknown alterations in cancer and to be used as a classification tool of different cancer types. In our laboratory, the combination of tissue proteomics and machine‐learning classified diffuse large B‐cell lymphoma depending on the cell of origin^81^, ^82^ and helped to identify most suitable cell line models for ovarian cancer research.^83^

But even before MS was able to capture nearly complete proteomes, several discoveries with translational potential have been made. For example, in 2007 a global survey of phosphotyrosine signaling in non‐small cell lung cancer (NSCLC) cell lines and tumors revealed unusually high activities of ALK and proto‐oncogene tyrosine‐protein kinase ROS (ROS).^84^ Follow‐up western blotting, RT‐PCR, and DNA sequencing in these samples revealed oncogenic ALK and ROS fusion proteins, including the fusion of ALK to echinoderm microtubule‐associated protein‐like 4 (EML4). Diagnostic tests designed to screen NSCLC tumors for the presence of rearrangements in ALK have consequently been commercialized for the selection of patients for treatment with Crizotinib.^85^

MS‐based proteomics may be particularly important in identifying targets in poorly mutated tumors such a pediatric ones. A recent study showed that malignant pediatric medulloblastomas do not show profound changes at the genomic and transcriptomic levels, however, using MS‐based phosphoproteomics, substantial differences were found.^86^ The proteomic data classified the medulloblastoma patients into subgroups, in which phosphorylation sites of MYC were associated with poor outcomes.

**Figure 2:**
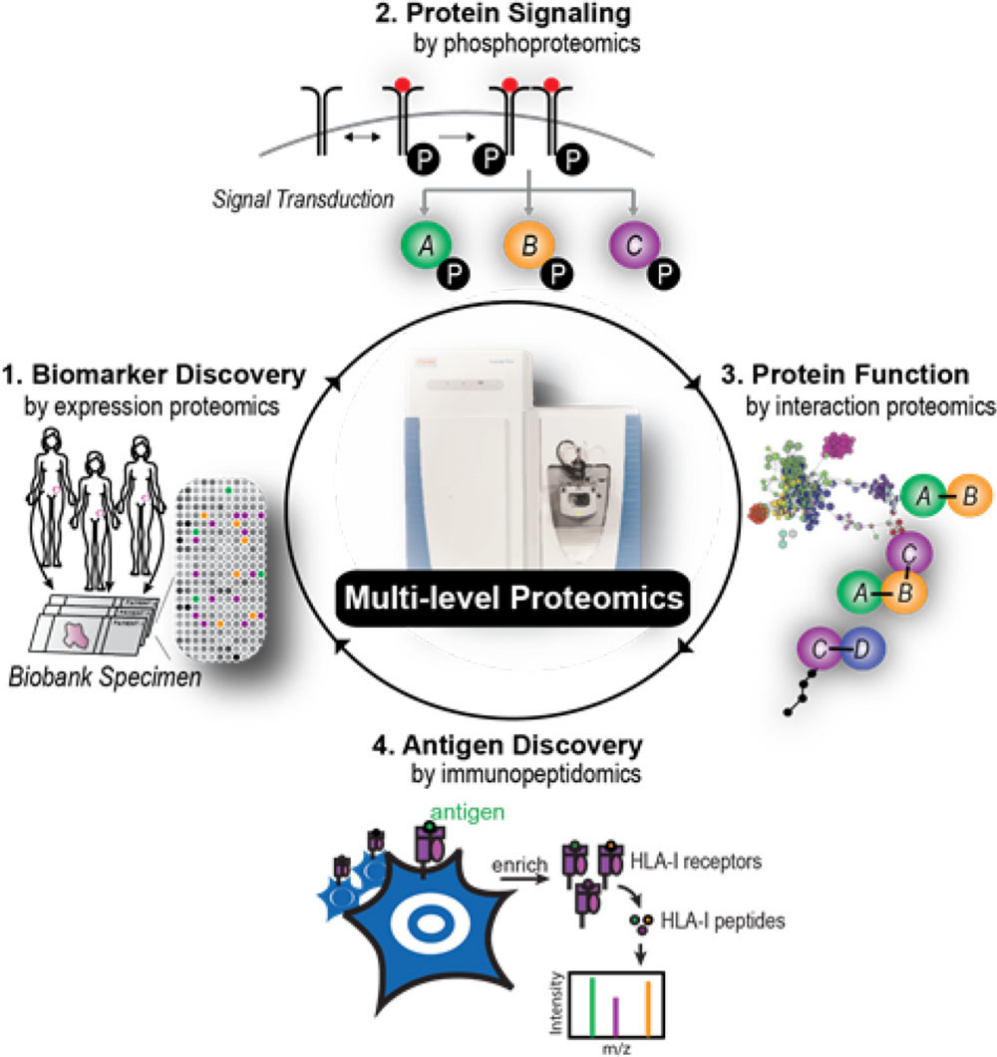
A multi‐level proteomics approach uncovers CT45 as a prognostic biomarker in ovarian cancer and sheds light on its biological role.^[75]^

Very recently, our group has discovered a first functional biomarker in solid tumors by integrating several proteomic layers, to study protein signaling, protein interactions, and antigen presentation.^76^ We started with the question why a small subset of women with high grade serous ovarian cancer have a long disease‐free survival after chemotherapy. Expression proteomics revealed that tumors of these women re‐expressed the cancer/testis antigen family 45 (CT45), which is normally not present in adult tissue. Nothing about the function of CT45 was known, but interaction proteomics^87^ showed that CT45 is associated with a protein complex involved in DNA damage signaling. Phosphoproteomics further demonstrated involvement of the Fanconi anemia signaling pathway. Interestingly, we found that CT45 likely has a dual mode of action. Through direct interaction with the protein phosphatase 4 complex, CT45 functions as a tumor‐intrinsic enhancer of chemosensitivity, rendering cells hypersensitive toward carboplatin‐based chemotherapy. Additionally, we identified CT45 as a naturally presented, cancer‐specific antigen recognized and targeted by patient‐derived cytotoxic T‐cells (**Figure** 2). Both of these findings are of direct clinical relevance and highlight the versatility of proteomics technology to identify novel treatment targets for chemo‐ or immunotherapeutic regimens.

Almost all tumors are heterogeneous and this presents an analytical and clinical challenge in oncology. So far this heterogeneity has almost exclusively been addressed at the oligonucleotide level. However, with the ever increasing sensitivity of MS‐based proteomics, it is now possible to separately analyze tumor versus stroma or using laser capture microdissection.^55^, ^88^, ^89^ In special circumstances, it is already possible to obtain relatively deep proteomes from less than 1000 cells.^32^ Current development should make it possible to analyze just a few hundred cells in FFPE material (Coscia et al., in preparation). In the future, MS may even be able to analyze individual cells, however, throughput would likely remain relatively low, due to the absence of bar‐coding.

## 6 MS as a Promising Tool for the Clinic

While MS‐based proteomics is already widely accepted as a discovery tool, recent advances in resolution, robustness, reproducibility, and speed now also allow envisioning the implementation of the technology directly in a clinical context. Please see **Table** 1 for an overview of promising applications of MS on clinical studies. Although much work remains to make the technology fully mature for “clinical‐level standards,” there are now promising developments toward its application in the clinic.^76^, ^90^, ^91^, ^92^, ^93^ Furthermore, proteomic data have been used to predict response to therapies in different tumor entities.^94^ So far, the greatest focus has been on the discovery and establishment of protein biomarkers in liquid biopsies, in particular blood plasma.^95^ Blood tests are of particular interest because of their noninvasive nature compared to invasive surgeries for obtaining biopsies of cancer tissues. Detection of protein biomarkers in blood, however, is particularly challenging due to the complexity of the plasma proteome and the high abundance range of proteins. These technological challenges as well as conceptual and study design issues have contributed to the near absence of novel, MS‐derived biomarkers in plasma.^51^ However, new MS workflows have recently been developed for fast and robust^79^, ^96^ or very deep measurements of plasma samples with promising results.^97^ Therefore, MS‐based proteomics might enable the future discovery of clinically actionable protein biomarker patterns in liquid biopsies and answer relevant clinical questions in a much more specific manner.

**Table 1:** Mass spectrometry‐based proteomic and phosphoproteomic clinical cancer studies

Publication	Cancer type	Sample type	PTM
Zhang et al., *Cancer Res*., 2004	Ovarian	Serum	—
Fung et al., *Clin. Chem*., 2010	Ovarian	Serum	—
Zhang et al., *Nature*, 2014	Colorectal	Tissue	—
Mertins et al., *Nature*, 2016	Breast	Tissue	+Phosphorylation
Yu et al., *J. Proteome Res*., 2016	Ovarian	Tissue	+Phosphorylation
Kim et al., *Nat. Commun*., 2016	Prostate	EPS‐Urine	—
Zhang et al., *Cell*, 2016	Ovarian	Tissue	+Phosphorylation acetylation
Wang et al., *Cell Chem. Biol*., 2018	Breast	Tissue	—
Doll et al., *Mol. Oncol*., 2018	Urachal	Tissue	—
Archer et al., *Cancer Cell*, 2018	Medulloblastoma	Tissue	+Phosphorylation acetylation
Coscia et al., *Cell*, 2018	Ovarian	Tissue	+Phosphorylation

As outlined above, tissues are already very amenable to MS‐based proteomics, and ongoing developments will make deep proteome coverage increasingly routine. Among other applications, this makes the technology applicable to “case studies” of single patients, a well‐established paradigm in medicine. Physicians usually treat patients that have been diagnosed with a defined cancer type with the same standard treatments and monitor the outcome. In case of nonresponse, toxicity or resistance, they move on to second or third line therapy. In the cancer genomics field, several companion diagnostic (CDx) tests now support physicians in identifying the appropriate therapeutic treatment for each patient individually.^98^, ^99^, ^100^ In particular, several trials have used sequencing for advanced or chemorefractory cancer patients, however, additional therapeutic benefit was modest.^16^, ^101^, ^102^ To our knowledge, no such efforts have been implemented on the basis of high‐resolution MS‐based cancer proteomics.

We reasoned that the recent technological advances, would make MS‐based proteomics a powerful tool for uncovering additional, personalized treatment options for chemorefractory patients. Given the time constraints in this situation, we developed a fast workflow, taking only 2.5 days from obtaining the sample to interpreted result, which is much faster than corresponding oligonucleotide‐based technology turnaround times. We employed this workflow for a patient with end‐stage urachal carcinoma patient, who had already received multiple standard chemotherapies for her primary and metastatic tumor.^80^ From the tumor and surrounding tissue, we discovered a set of upregulated proteins, whose biological roles reflected tumor biology. Among these, the epigenetic regulator lysine specific histone demethylase 1 (LSD1) caught our attention, because it is a promising therapeutic target and the focus of current drug development efforts in several large pharmaceutical companies.^103^ Complementing the proteomic data with NGS for mutation calling and a newly developed “clinical knowledge graph” that integrates vast amounts of diverse information, helped to guide the therapy decision. Based on this data, the tumor board approved treatment of the patient with a drug targeting LSD1. We have already applied our workflow to a number of other chemorefractory patients and we envision that it can be broadly applied in the future.

## 7 Proteogenomics: Combining Genomics and Mass Spectrometry‐Based Proteomics Data

The development of personalized medicine will require a closer integration of omic‐based approaches, in particular genomic and proteomic data, and clinical data.^104^ To this end, similar to TCGA, the National Cancer Institute has launched a clinical proteomic tumor analysis consortium (CPTAC) that aims to develop and standardize workflows to systematically identify and characterize cancer‐relevant proteins and their underlying biological pathways. The combination of proteomic and genomic data, termed proteogenomics, suggested novel proteomic tumor subtypes associated with clinical outcome.^33^, ^34^, ^35^ Of note, protein levels could not be predicted from genomic or transcriptomic data, emphasizing the importance of studying the actual molecular actors within a cellular system. A link between protein acetylation and phosphorylation to deficiencies in homologous recombination was identified in ovarian cancer patients.^35^ Since homologous recombination deficiency is associated with susceptibility to PARP inhibitors and improved survival, this suggests a potential patient stratification for therapies.^105^, ^106^ Large number of kinases, such as ribosomal protein S6 kinase alpha‐5 (RPS6KA5) and eIF‐2‐alpha kinase GCN2 (EIF2AK4) were also identified, which are affected by common PIK3CA mutations. Furthermore, cellular tumor antigen p53 (P53)‐mutation‐associated phosphopeptides revealed previously unknown functionalities, such as the regulation of the kinases serine/threonine‐protein kinase greatwall (MASTL) and eukaryotic elongation factor 2 kinase (EEF2K).

Since transferases, including kinases^107^ and epigenetic regulators,^10^ are among the most frequently mutated proteins in cancer, it is important to examine the interplay between PTMs and genomic alterations. Bioinformatics resources such as PhosphoSitePlus already integrate PTM sites along with somatic mutations and cancer‐associated germline variants, illustrating the importance of uniting genomics with proteomics to better understand the underlying molecular mechanisms of cancer.^108^, ^109^

While, the driving role of receptor tyrosine kinases, as predicted from their genomic mutation profile, was validated in a patient‐derived xenograft model of breast cancer, genomics did not explain the occurrence and role of the phosphorylation events.^110^ This shows that druggable phosphorylation events can readily be uncovered using proteomics. Proteogenomics is particularly promising in highly mutated tumors, such as melanoma.^111^ As MS‐based proteomics generally requires a reference database to match fragmentation spectra, it can be challenging to identify mutated peptides that are not present in this database. Thus, sequencing the patient and tumor genomes and creating a personalized reference proteomic reference database enables the identification of individualized mutated peptides, if they are expressed at the protein level. Putative neoantigens have already been identified using proteogenomics and proven to be successful as vaccines promoting an antitumor immune response and they are currently approaching clinical trials.^112^, ^113^ A RNA‐affinity proteogenomic approach also distinguished breast cancer patients with different clinical outcomes and thus helped to guide therapeutic decisions.^114^ These studies highlight the added value of proteogenomic analysis over using genomics‐driven approaches only in the clinical characterization of cancers.

## 8 Conclusion and Outlook

In 2017 the FDA approved pembrolizumab for the treatment of any unresectable or metastatic solid tumor with microsatellite instability.^115^ This was the first time that a cancer drug was introduced based on genetics rather than tumor site or tissue type. More generally, omics technologies are transforming the process of target discovery in pharmaceutical companies, as entire perturbed molecular landscapes can now be objectively and comprehensively mined. That said, the clinical implementation of NGS technologies, except for mutation calling, has not gained the same momentum yet.

We are still far from understanding the entire complexity of cancer. The most evident challenge for establishing omics technology as routine methods into the clinic is the complex biology of cancer. Each tumor is unique, and requires multiple omics layers to characterize its molecular profile as an interplay of these layers. In this perspective, we have argued that the value of molecular characterization for diagnoses and therapies of tumors is now indisputable. Proteomics, including the identification of PTMs will indeed provide a crucial missing piece in many biological conundrums in oncology. So far MS‐based proteomics has been held back by immature technology, but this is clearly changing rapidly.

**Figure 3:**
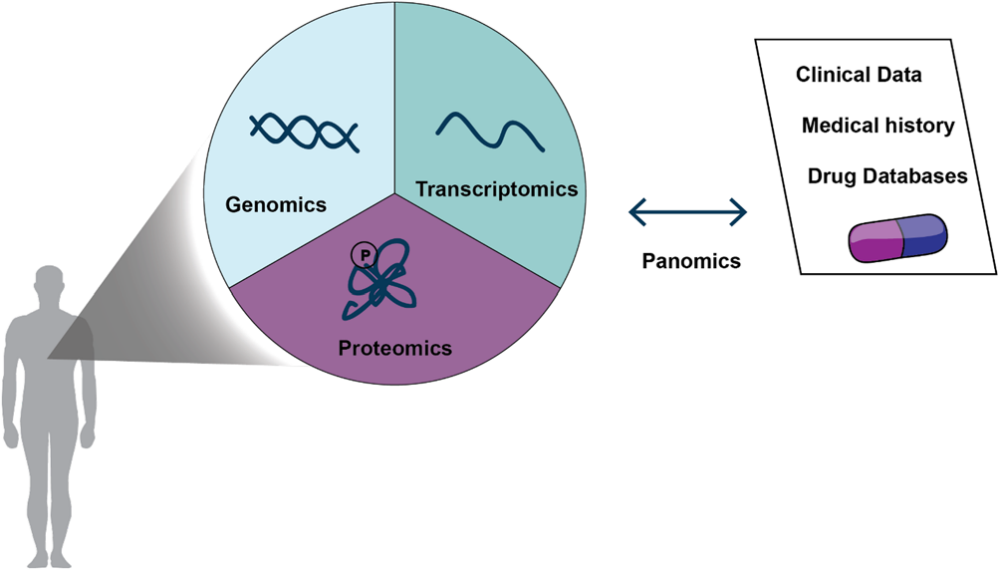
Panomics‐based approach integrating genomic, transcriptomic, and proteomic data with clinical data to better identify therapeutic treatments for cancer patients.

One of the challenges of bringing MS‐based proteomics into the clinic is the associated cost and the cutting‐edge expertise that is still required. However, today top‐end mass spectrometers cost half a million to a million dollars and this is not too different from high end imaging equipment that is routinely used in oncology. Arguably, with robust instrumentation and high utilization, costs could even be significantly lower than those technologies. However, the expertise to operate these instruments and to analyze the data is still needed. Although this will be the case for the next future, we hope that more robust instrumentation, broad training, and standardization will eventually change this picture. In our own laboratory, the analysis of cancer tissues has become quite robust, being performed in an automated manner on a robotic platform. In this way, 12 cancer proteomes can already be measured per mass spectrometer per day and improvements currently underway in the entire LC MS/MS workflow may soon increase the throughput and robustness further.^64^, ^65^ We envision a future in which genomics, transcriptomics, proteomics, and other omics and clinical data will go hand in hand to define the best treatment for the patient (**Figure** 3).

However, there are still many challenges ahead. Assuming that the associated avalanche of data can be standardized, processed, stored, and securely shared, omics‐based personalized medicine will still face the challenge of false positives or negatives. For example, different “state‐of‐the‐art” computational methods predicting the effects of missense mutations yield inconsistent results.^116^ It is unacceptable that the life of a patient should hinge on the insufficient development of computational classifiers. The bioinformatics community recognizes this challenge, and multiple efforts aim to standardize, tune, or combine computational methods to enhance crucial applications such as mutation calling.^117^ In general, the increasing use of artificial intelligence will further empower personalized cancer medicine by enabling the usage of large data.^118^

As outlined above, pioneering studies have demonstrated the potential of MS‐based proteomics for biomarker discovery and clinical applications. Proteomics already created the possibility for medical doctors and scientists to open up new treatment options for end stage cancer patients by identifying actionable therapeutic targets. As the MS technology becomes increasingly sensitive, the analysis of minute amounts of laser capture microdissected tissues and even close to single cell analysis will become increasingly realistic. Furthermore, the automation of the workflows will enable the characterization of much larger patient cohorts and will lead to the identification of clinically relevant biomarkers.

This approach will not only require many technological advances but also a cultural shift on many levels—in regulatory agencies, in pharmaceutical companies and, most of all, in the clinic. It will require an openness toward substantially new approaches of doctors, as well as health care providers—not least insurance providers.^119^ More clinical data are needed to evaluate clinical utility of omics testing, so that insurers can make decisions about coverage. Large‐scale data collection, however, is impeded by uncertain reimbursement policies. This dilemma has to be resolved to establish omics in the clinic.

By combining large scale and individual genomic, (phospho)proteomic and clinical data we envision a bright future by providing fundamental insights into the underlying molecular mechanisms of cancer and offering novel therapeutic strategies in the field of personalized medicine.

## Conflict of Interest

The authors declare no conflict of interest.
